# CsNAC17 enhances resistance to *Colletotrichum gloeosporioides* by interacting with CsbHLH62 in *Camellia sinensis*

**DOI:** 10.1093/hr/uhae295

**Published:** 2024-10-14

**Authors:** Rui Han, Huiling Mei, Qiwei Huang, Cunqiang Ma, Yuxin Zhao, Anburaj Jeyaraj, Jing Zhuang, Yuhua Wang, Xuan Chen, Shujing Liu, Xinghui Li

**Affiliations:** College of Horticulture, Nanjing Agricultural University, Nanjing 210095, China; College of Resources and Environmental Sciences, Nanjing Agricultural University, Nanjing 210095, China; College of Resources and Environmental Sciences, Nanjing Agricultural University, Nanjing 210095, China; College of Horticulture, Nanjing Agricultural University, Nanjing 210095, China; College of Horticulture, Nanjing Agricultural University, Nanjing 210095, China; College of Horticulture, Nanjing Agricultural University, Nanjing 210095, China; College of Horticulture, Nanjing Agricultural University, Nanjing 210095, China; College of Horticulture, Nanjing Agricultural University, Nanjing 210095, China; College of Horticulture, Nanjing Agricultural University, Nanjing 210095, China; College of Horticulture, Nanjing Agricultural University, Nanjing 210095, China; Tea Research Institute, Nanjing Agricultural University, Nanjing 210095, China

## Abstract

The pathogen *Colletotrichum gloeosporioides* causes anthracnose, a serious threat to tea trees around the world, particularly in warm and humid regions. RNA-Seq data have previously indicated NAC transcription factors are involved in anthracnose resistance, but underlying mechanisms remain unclear. The BiFC, Split-LUC, and Co-IP assays validated the interaction between CsbHLH62 and CsNAC17 identified through yeast two-hybrid (Y2H) screening. *CsNAC17* or *CsbHLH62* overexpression enhanced anthracnose resistance, as well as enhanced levels of H_2_O_2_, hypersensitivity, and cell death in *Nicotiana benthamiana*. The NBS-LRR gene *CsRPM1* is regulated by CsNAC17 by binding directly to its promoter (i.e. CACG, CATGTG), while CsbHLH62 facilitates CsNAC17’s binding and increases transcriptional activity of *CsRPM1*. Additionally, transient silencing of *CsNAC17* and *CsbHLH62* in tea plant leaves using the virus-induced gene silencing (VIGS) system resulted in decreased resistance to anthracnose. Conversely, transient overexpression of *CsNAC17* and *CsbHLH62* in tea leaves significantly enhanced the resistance against anthracnose. Based on these results, it appears that CsbHLH62 facilitates the activity of CsNAC17 on *CsRPM1*, contributing to increased anthracnose resistance.

## Introduction

*Colletotrichum* sp. causes anthracnose, a damaging disease that threatens *Camellia sinensis* (L.) O. Kuntze cultivation [1]. While the pathogen primarily infects young tissues, symptoms are more pronounced on mature leaves due to extended incubation periods [2]. The deleterious effects of anthracnose on yield and quality underscores the need for effective prevention and control measures. Presently, on account of commonly used chemical agents that raise concerns about potential threats to food security [3], the study on related disease-resistance genes offers a foundation for enhancing disease resistance in tea plants via breeding or genetic modification.

Plants evolve resistance genes that specifically recognize effectors produced by pathogens. The NBS-LRR gene family, a significant class of plant R genes, is commonly engaged with the ETI response, which defends against pathogen infection [4]. Currently, in *Arabidopsis* ~200 NBS-LRR genes have been cloned, with *RPS2* and *RPM1* being the most archetypal [5]. RPM1, a CC-NBS-LRR protein kinase, specifically recognizes pathogen-released effectors, thereby triggering both ETI and hypersensitive response (HR) to resist diseases [6]. Through direct interaction with OsWRKY19, overexpression of the rice *RPM1* homolog, *OsRLR1*, induces HR in rice leaves and enhances resistance [7]. Following *Exobasidium vexans* infection in tea plant, both *RPM1* and *RPS* show significant expression [8].

A variety of NAC transcription factors are associated with the advancement of sidelong roots [9], the regulation of seed germination genes and albino phenotype [10, 11], and the modulation of senescence-induced cell death in plants [12]. Additionally, NAC proteins modulate reactive oxygen species (ROS) accumulation and regulate resistance gene expression because of abiotic and biotic anxieties. *BnaNAC55* and *BnaNAC103* induce ROS accumulation and cell death analogous to HR because of fungus in *Brassica napus* [13]. Overexpression of *VvNAC72* in grape elevates cytotoxic methylglyoxal (MG) content and ROS levels, consequently enhancing obstruction against *Plasmopara viticola* [14]. *VvNAC72* suppresses *VvGLYI-4* transcription by directly binding to its promoter region through the CACGTG element. *ONAC083* binds directly to the ACGCAA element, activating *OsRFPH2–6*, *OsTrx1*, and *OsPUP4*, negatively regulating rice immunity against PTI caused by *Magnaporthe oryzae* [15]. In tea plants, NAC expression levels are associated with metabolic regulation [16, 17] and anthracnose resistance [18].

There is evidence that bHLH transcription factors are involved in pathogen responses in plants. Powdery mildew resistance is boosted by transient overexpression of *MdbHLH093* in apple leaves [19]. SlJIG, a direct target of MYC2, structures a functional module with MYC2 and confers resistance against *Botrytis cinerea* [20]. Overexpression of *GhPAS1*, a bHLH TF, presents enhanced resistance against *Verticillium dahlia*. Specifically, *GhPAS1* increases promoter activity of *GhERF14* and *GhPER64*, which are straightforwardly associated with *Verticillium* resistance. Additionally, *GhPAS1* enhances resistance by directing lignin biosynthesis and defense-related genes [21]. The OsbHLH6 protein controls the salicylic acid and jasmonic acid signaling pathways by moving between the nucleus and cytoplasm, providing disease resistance [22]. Furthermore, OE-*OsbHLH057* significantly enhances rice disease resistance. In particular, OsbHLH057 binds to the AATCA motif within *Os2H16*, which exhibits an antifungal response [23]. *VabHLH137* overexpression significantly enhanced resistance against *Colletotrichum gloeosporioides* by inducing the upregulation of *VaLAR2*, thereby promoting the biosynthesis of PA and augmenting defense mechanisms in grape callus [24]. The bHLH factors primarily form complexes with MYB TFs in tea plants, regulating metabolic synthesis of flavonoids [25], catechins [26], flavan-3-ols [27], and EGCG3'Me [28]. However, there is a lack of investigation in the composition of bHLH complex in plants’ disease response.

As shown in our previous transcriptome analysis, Zhongcha108 (ZC108), as compared to Longjing43 (LJ43), exhibits higher expression of the NAC transcription factor *CsNAC17* [18]. In our study, we discovered an interaction between CsNAC17 and the transcription factor, *CsbHLH62*. Overexpressing *CsNAC17* or *CsbHLH62* significantly inhibits the development of *C. gloeosporioides* in both tobacco and tea plant leaves. Additionally, *CsNAC17* directly regulates the *CsRPM1* expression, and the interaction between CsbHLH62 and CsNAC17 promotes *CsRPM1* expression following anthracnose infection. These discoveries feature the crucial job of CsbHLH62 and CsNAC17 collaboration in controlling anthracnose obstruction in tea plants, offering potential implications for developing resistant tea seeds.

## Results

### Characterization of *CsNAC17* in response to *C. gloeosporioides*

**Figure 1:**
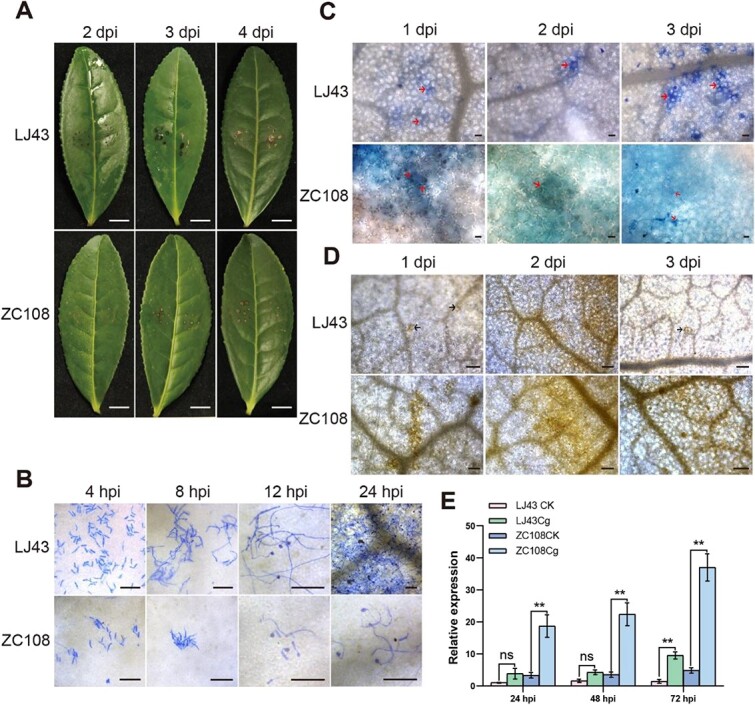
Histochemical observation of ‘Longjing 43’ (LJ43) and ‘Zhongcha 108’ (ZC108) inoculated with *C. gloeosporioides*. **(A)** Disease symptom on leaves after *C. gloeosporioides* inoculation at 2, 3, and 4 dpi. Scale bar = 1 cm. (**B)** The development of *C. gloeosporioides* on ‘Longjing 43’ and ‘Zhongcha108’ staining with aniline blue at 4, 8, 12, and 24 hpi. Aniline blue can stain conidium and hyphae. Scale bar = 50 μm. (**C)** The hypersensitive response lesions in the leaves of ‘Longjing 43’ and ‘Zhongcha108’ following *C. gloeosporioides* infection were visualized through trypan blue staining at 1, 2, and 3 dpi. Trypan blue can stain cell death. Arrows indicate hypersensitive response lesions. Scale bar = 50 μm. (**D)** H_2_O_2_ detection by DAB staining at 1, 2, and 3 dpi. Arrows indicate H_2_O_2_ accumulation. Scale bar = 200 μm. (**E)** Relative expression of *CsNAC17* in ‘Longjing 43’ and ‘Zhongcha108’ after *C. gloeosporioides* inoculation at 24, 48, and 72 hpi. RT-qPCR data were introduced as mean values ± standard deviation (**, *P* <0.01; *n* = 3).

The pathogenicity test demonstrated that the characteristic anthracnose brown spot manifested on the ‘LJ43’ leaves during pathogen infection at 3 and 4 days post-inoculation (dpi), but with no obvious disease symptoms on the ‘ZC108’ leaves (Fig. 1a). Aniline blue staining techniques was applied to examine the infection structures of the fungi *C. gloeosporioides* in tea plant leaf tissues, which revealed that the development of *C. gloeosporioides* on ‘LJ43’ leaves was faster than that on ‘ZC108’ (Fig. 1b). Trypan blue staining revealed apparent significant HR formation on ‘ZC108’ leaves during *C. gloeosporioides* infection, while no such response was observed on ‘LJ43’ leaves. Furthermore, ‘ZC108’ leaves showed epidermal cell necrosis, whereas only mesophyll cell necrosis was noted in ‘LJ43’ leaves, but no evidence of epidermal cell necrosis (Fig. 1c). The production and accumulation of H_2_O_2_ was observed by DAB staining. The results demonstrated a significantly greater accumulation of H_2_O_2_ on ‘ZC108’ leaves than that on ‘LJ43’ leaves (Fig. 1d). These findings confirmed that ‘ZC108’ is more resistant to *C. gloeosporioides* compared to ‘LJ43’. Combined with our previous transcriptome data in ZC108 after *C. gloeosporioides* infection [29], we proposed *CsNAC17* as an important resistance factor that was further proved by real-time quantitative polymerase chain reaction (RT-qPCR). The *CsNAC17* transcripts were upregulated in ‘ZC108’ at 24–72 h post-inoculation (hpi), reaching its peak expression at 72 h. However, it was upregulated at 72 hpi in ‘LJ43’ and no significant difference at 24–48 hpi (Fig. 1e).

**Figure 2:**
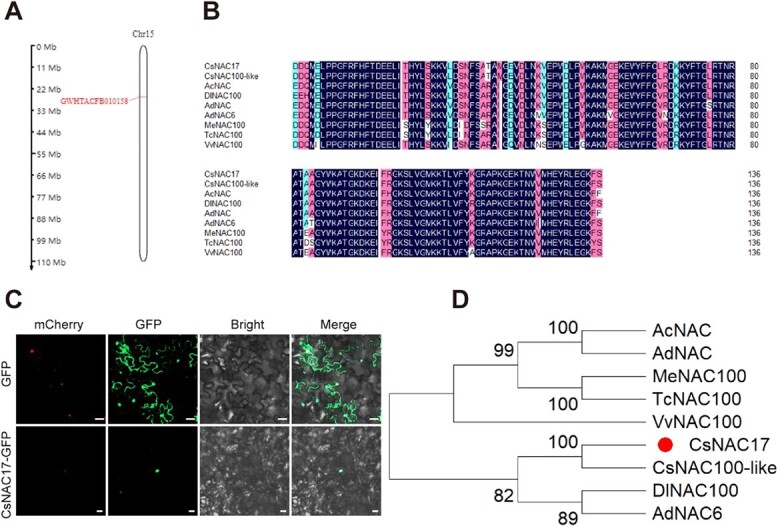
Sequence analysis of CsNAC17 isolated from ‘Zhongcha108’. (**A)** Chromosome location of *CsNAC17*. CsNAC17 is located on chromosome 15, from position 31 011 935 to 31 013 995. (**B)** Protein sequence alignment of conserved domain of CsNAC17. CsNAC100-like (*C. sinensis*, XP_028056118); AcNAC (*Actinidia chinensis*, PSR89671); DlNAC100 (*Diospyros lotus*, XP_052179391); AdNAC (*Actinidia deliciosa*, QFG73549); AdNAC6 (*A. deliciosa*, AZL19352); MeNAC100 (*Manihot esculenta*, XP_021614789); TcNAC100 (*Theobroma cacao*, XP_017972125); VvNAC100 (*Vitis vinifera*, XP_002284825). (**C)** CsNAC17 localization in tobacco leaves. mCherry was used as a positive nuclear marker. Bar = 20 μm. (**D)** Phylogenetic analysis of CsNAC17. The circle indicates CsNAC17.

CsNAC17 is located on chromosome 15 and encodes a protein of 357 amino acids with a molecular weight of 40.278 kDa and an isoelectric point of 8.23 (Fig. 2a). CsNAC17, with NAM conserved domain, belongs to the NAM protein family. Protein sequence alignment revealed the highest similarity of CsNAC17 with DlNAC100 and AdNAC6 (Fig. 2b). Heterologous expression of CsNAC17-GFP fusion protein in tobacco indicates nuclear localization (Fig. 2c). The highest homology was found between *CsNAC17* and *DlNAC100* and *AdNAC6* based on phylogenetic analysis (Fig. 2d). A comparison of the CsNAC17 protein sequence with other *C. sinensis* varieties demonstrated a 100% similarity between ‘Tieguanyin’ and ‘LJ43’ (Supplemental Fig. S1a). The yeast two-hybrid (Y2H) analysis confirmed that the transcriptional activation activity of CsNAC17 could be inhibited on SD/−T/X/A (200 ng ml^−1^) medium (Supplemental Fig. S1b). The hydrophilic–hydrophobic distribution curve revealed a substantial hydrophobic region in its peptide chain (Supplemental Fig. S1c). The tertiary structure of CsNAC17 protein was predicted using the SWISS-MODEL database (https://swissmodel.expasy.org/) (Fig. S1d).

### *CsNAC17* is a positive regulator of anthracnose resistance in *Nicotiana benthamiana*

**Figure 3:**
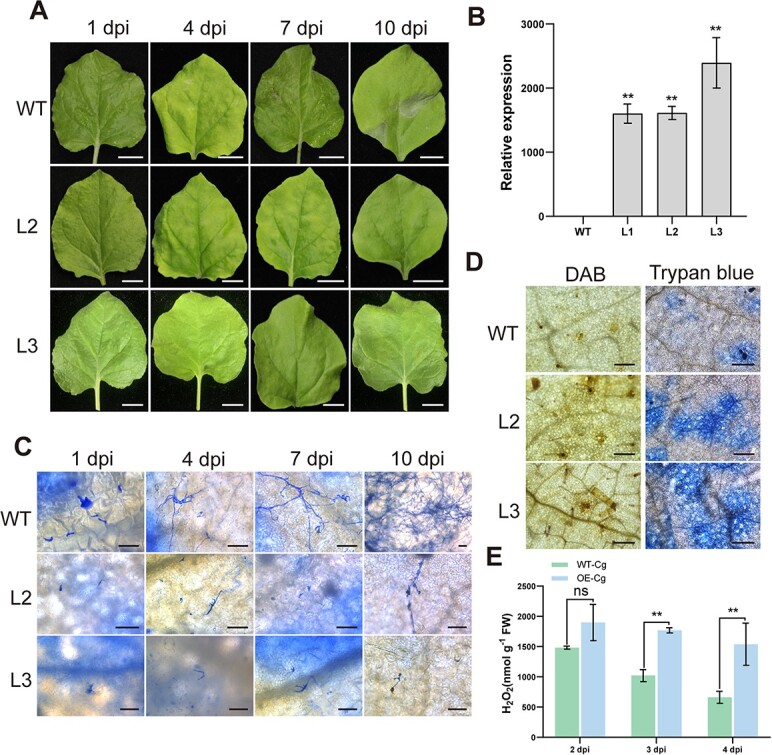
Overexpressing *CsNAC17* in tobacco enhances resistance to anthracnose. **(A)** Inoculations with *C. gloeosporioides for* 1, 4, 7, and 10 dpi with transgenic lines (L2, L3) and WT. Scale bar = 1 cm. (**B)** Confirmation of OE lines by RT-qPCR analysis. RT-qPCR data were introduced as mean values ± standard deviation (**, *P* <0.01; *n* = 3). (**C)** The mycelial growth of *CsNAC17*-OE lines and WT was stained with trypan blue. Scale bar = 50 μm. (**D)** Leaves infected with *C. gloeosporioides* for 4 dpi were stained using trypan blue and DAB. Trypan blue was utilized to stain cellular death, while DAB was employed to detect the presence of H_2_O_2_. Scale bar = 200 μm. (**E)** Measurement of the H_2_O_2_ content in leaves after infection with *C. gloeosporioides* at 2, 3, and 4 dpi. Data with three biological replicates were introduced as mean values ± standard deviation (**, *P* <0.01).

Tobacco lines overexpressing *CsNAC17* (OE-*CsNAC17*) were constructed to investigate its role in fungal resistance. Following independent transformation (Fig. 3b; Supplemental Fig. S2a), homozygous transgenic plants were identified and three lines (i.e. L2, L3) with elevated *CsNAC17* expression levels were selected. Inoculation tests of OE-*CsNAC17* tobacco T3 generation lines and wild-type (WT) by *C. gloeosporioides* revealed small water-soaking spots in WT leaves at 4 dpi, while transgenic plant leaves had no obvious changes. Notably, a gradual increase in lesion area occurred in WT leaves between 7 and 10 dpi, whereas transgenic leaves exhibited no such alterations (Fig. 3a). Trypan blue staining illustrated limited or just initiated germination of *C. gloeosporioides* spores in both WT and transgenic lines at 1 dpi. The hyphae of the pathogen in the WT leaves were notably longer, while transgenic lines displayed shorter hyphae at 4 dpi (Fig. 3c). The hyphae congregated in the WT leaves, whereas the hyphae growth in the transgenic lines was hindered, with the haustoria being dried and shriveled from 7 dpi (Fig. 3c). WT leaves harbored numerous conidia, with hyphae covering the entire leaves, while transgenic lines exhibited no significant changes in pathogens at 10 dpi (Fig. 3c). DAB staining revealed a higher H_2_O_2_ accumulation in the transgenic leaves compared to the WT at 4 dpi (Fig. 3d), which was consistent with the observed change in H_2_O_2_ content determined by a kit (Fig. 3e), suggesting a stronger HR response occurred in the OE-*CsNAC17* lines compared to the WT.

### CsNAC17 directly interacts with CsbHLH62

**Figure 4:**
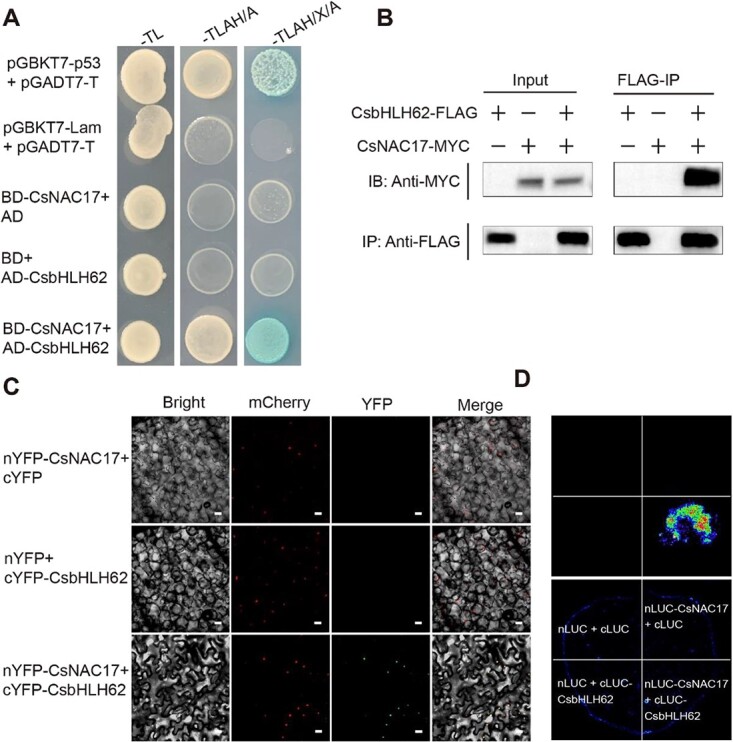
Interaction analysis of CsNAC17 and CsbHLH62. **(A)** Y2H assay showing the interaction between CsNAC17 and CsbHLH62 in yeast cells. pGBKT7-p53 + pGADT7-T and pGBKT7-Lam + pGADT7-T, BD-CsNAC17+ AD, BD + AD-CsbHLH62 served as positive and negative controls, respectively. Abbreviations: SD/−Trp-Leu/X; SD/−Trp-Leu-His-Ade/AbA; SD/−Trp-Leu-His-Ade/X-α-Gal/aureobasidin A; AD, pGADT7; BD, pGBKT7. (**B)** Co-IP assays showing the interaction between CsNAC17 and CsbHLH62. (**C)** CsNAC17 and CsbHLH62 interact in the nucleus of tobacco in a bimolecular fluorescence complementation assay. Nuclei were counterstained with Marker mCherry. nYFP-CsNAC17 + cYFP and nYFP + cYFP- CsbHLH62 served as negative controls. Scale bar = 20 μm. (**D)** Split-LUC assay showing the interaction between CsNAC17 and CsbHLH62. The controls were: nLUC + cLUC; nLUC- CsNAC17 + cLUC; nLUC + cLUC- CsbHLH62. Luminescence intensity showed the interaction of CsNAC17 and CsbHLH62 in tobacco.

CsNAC17 resistance mechanism was investigated using the Y2H assay. BD-CsNAC17 was used as bait for screening the cDNA library and eight clones were found (Table S3). Among them, CsbHLH62, a transcription factor belonging to the bHLH family, known for its role in plant disease resistance, was selected for further analysis [30]. As with the positive control, yeast colonies harboring BD-CsNAC17 and AD-CsbHLH62 grew on SD/−Ade-His-Leu-Trp medium and displayed a blue coloration (Fig. 4a). The results confirmed the specific interaction between CsNAC17 and CsbHLH62 in yeast cells.

The interaction between CsNAC17 and CsbHLH62 was further validated through co-immunoprecipitation experiments in tobacco leaves employing CsNAC17-MYC and CsbHLH62-FLAG (Fig. 4b). The bimolecular fluorescence complementation (BiFC) analysis in tobacco leaves revealed a fluorescent signal in the nucleus resulting from the combination of nYFP-CsNAC17 and cYFP-CsbHLH62, as opposed to the control sample (Fig. 4c). Subsequent split-luciferase assay demonstrated an exclusive fluorescent signal in the combination of nLUC-CsNAC17 and cLUC-CsbHLH62 by CCD imaging after 48 h of co-transformation (Fig. 4d), supporting the interaction between CsNAC17 and CsbHLH62.

### Expression of *CsbHLH62* heterologously enhances anthracnose resistance in *N. benthamiana*

**Figure 5:**
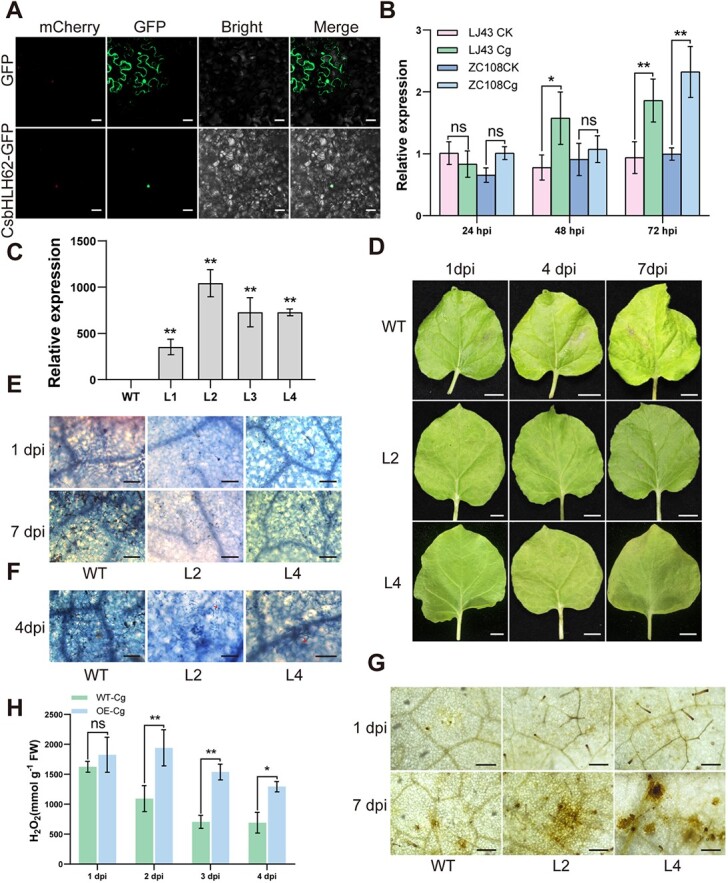
*CsbHLH62* transgenic tobacco exhibits enhanced resistance to anthracnose. (**A)** CsbHLH62 is localized in the nucleus of tobacco. mCherry was used as a positive nuclear marker. Scale Bar = 20 μm. (**B)***CsbHLH62* expression in leaves of ‘Longjing 43 (LJ43)’ and ‘Zhongcha 108 (ZC108)’ after *C. gloeosporioides* inoculation at 24, 48, and 72 hpi. RT-qPCR data were introduced as mean values ± standard deviation (*, *P* <0.05; **, *P* <0.01, *n* = 3). (**C)** Confirmation of OE lines by RT-qPCR analysis. RT-qPCR data were introduced as mean values ± standard deviation (**, *P* <0.01; *n* = 3). (**D)** Phenotypes of transgenic lines and WT after inoculation with *C. gloeosporioides* for 1, 4, 7, and 10 dpi. Scale bar = 1 cm. (**E)***CsbHLH62*-OE lines (L2, L4) and WT stained with trypan blue at 1 and 7 dpi. Scale bar = 100 μm. (**F)** Leaves infected with *C. gloeosporioides* for 4 dpi were stained with trypan blue. Arrows indicate cellular death. Scale bar = 100 μm. (**G)** DAB staining for H_2_O_2_ detection after infection with *C. gloeosporioides* for 1 and 7 dpi. Scale bar = 200 μm. **(H)** Measurement of the H_2_O_2_ content in *CsRPM1* OE lines and WT leaves after infection with *C. gloeosporioides* at 24 and 48 hpi. Data were introduced as mean values ± standard deviation (*, *P* <0.05; **, *P* <0.01; *n* = 3).

The *CsbHLH62* CDS from ‘ZC108’ was cloned and sequenced. Sequence analysis revealed only one amino acid variation, with a remarkable 99.8% similarity between ‘ZC108’ and ‘LJ43’ (Fig. S1e). *CsbHLH62* possessed an ORF of 1521 bp and was localized in the nucleus (Fig. 5a). RT-qPCR analysis revealed significant upregulation of *CsbHLH62* transcription both in ‘LJ43’ and ‘ZC108’ leaves at 72 hpi with *C. gloeosporioides* (Fig. 5b). Subsequently, tobacco lines overexpressing *CsbHLH62* (OE-*CsbHLH62*) were established to select high-expression lines L2 and L4 for further investigation (Fig. 5c; Supplemental Fig. S2b). The morphological analysis demonstrated that WT exhibited lesions 4 and 7 dpi, while the transgenic lines displayed no obvious symptoms (Fig. 5d). Trypan blue staining at 1 dpi revealed no significant disparity in leaf morphology between OE-*CsbHLH62* and the control, whereas a notable distinction was observed at 7 dpi. OE-*CsbHLH62* had limited hyphae compared to extensive coverage on WT leaves (Fig. 5e). After 4 dpi, we observed an obvious HR in OE-*CsbHLH62* lines, which was absent in the WT (Fig. 5f). Moreover, DAB staining indicated higher H_2_O_2_ accumulation in infected OE-*CsbHLH62* leaves compared to WT at 1 and 7 dpi (Fig. 5g). The H_2_O_2_ content indicated that H_2_O_2_ accumulation in OE-*CsbHLH62* lines was higher than that in WT after infection with *C. gloeosporioides* (Fig. 5h), suggesting that overexpressing *CsbHLH62* in tobacco enhanced defense response and resistance to anthracnose.

### CsbHLH62 enhances the transcriptional activity of *CsRPM1* by interacting with CsNAC17

**Figure 6:**
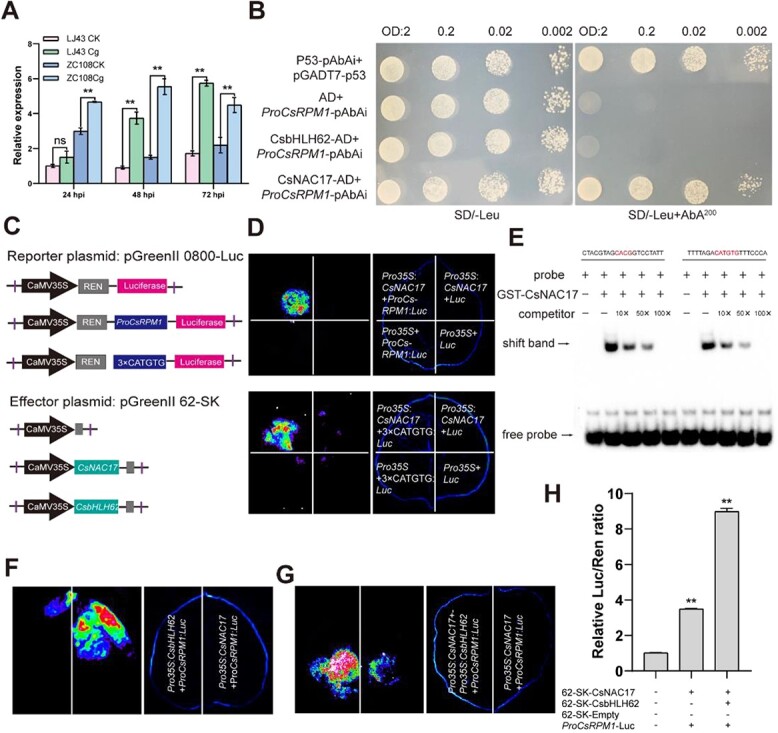
CsbHLH62 enhances the transcriptional levels of *CsRPM1* by interacting with CsNAC17. (**A)** CsRPM1 expression after inoculating ‘Longjing 43 (LJ43)’ and ‘Zhongcha 108 (ZC108)’ leaves with *C. gloeosporioides* at 24, 48, and 72 hpi. RT-qPCR data were introduced as mean values ± standard deviation (**, *P* <0.01; *n* = 3). (**B)** CsNAC17 binds to the promoters of *CsRPM1*, whereas CsbHLH62 cannot on SD/−Leu media containing 200 ng ml^−1^ Aureobasidin A. p53-pAbAi + pGADT7-p53 and AD + *ProCsRPM1*-pAbAi served as negative controls. Abbreviations: AD, pGADT7. **(C)** A diagram of the reporter and effector constructs for dual-luciferase assay. **(D)** Luciferase assay shows that CsNAC17 activates *CsRPM1* by binding to NACRS. The controls were: *Pro35S: CsNAC17* + Luc; *Pro35S* + *ProCsRPM1*: Luc; *Pro35S* + Luc. (**E)** CsNAC17 could specifically bind the NACRS (CATGTG, CACG) element of the *CsRPM1* promoter, as revealed using electrophoretic mobility shift assay. CACG and CATGTG indicate the binding site. **(F)** Luciferase (Luc) assay showed that CsbHLH62 can’t activate promoter activities of *CsRPM1*. *Pro35S: CsNAC17* + *ProCsRPM1*: Luc served as positive controls. (**G)** Luciferase (Luc) assay showed that CsNAC17 and CsbHLH62 co-transformed activate promoter activities of *CsRPM1*. The regulation of *ProCsRPM1* by CsNAC17 was further enhanced after the formation of heterodimers between CsbHLH62 and CsNAC17 proteins. (**H)** Luciferase (Luc) assay indicated that CsNAC17 increases *CsRPM1* promoter activity. CsNAC17 co-expressed with CsbHLH62 showed stronger activation of the *CsRPM1* promoter. The empty vector 62- SK and the *CsRPM1* promoter were set as 1. In the data, the mean ± standard deviation is represented (**, *P* <0.01; *n* = 3).

In tea plants, transient silencing of the *CsRPM1* gene following infection with *C. camelliae* results in significantly increased lesions, suggesting that the gene positively regulates tea plant resistance to *C. camelliae* [31]. Our study investigated the capability of *CsRPM1* because of *C. gloeosporioides*, revealing a significant upregulation in the transcription levels from 24 to 72 hpi in ‘LJ43’ and ‘ZC108’ leaves (Fig. 6a). *CsRPM1* promoter contains the NACRS (CATGTG, CACG) sequence (Supplemental Fig. S2c and e), which can be bound by the NAC proteins. Therefore, we predicted that CsNAC17 may regulate *CsRPM1* to enhance anthracnose resistance.

To test our hypothesis, we isolated the *CsRPM1* promoter (Supplemental Fig. S2c) from ‘ZC108’ genomic DNA. Employing the Y1H assay in yeast, we verified CsNAC17 interaction with the *CsRPM1* promoter. CsNAC17 directly binds to the *CsRPM1* promoter after determining a minimum inhibitory AbA concentration (200 ng ml^−1^) (Supplemental Fig. S2d; Fig. 6b). Additionally, the *ProCsRPM1* and NACRS elements were also fused to the luciferase reporter and co-transformed into tobacco epidermal cells together with the *Pro35S:CsNAC17* effector (Fig. 6c). CCD imaging revealed that CsNAC17 could positively regulate the expression of *CsRPM1* (Fig. 6d). Moreover, electrophoretic mobility shift assay (EMSA) was performed to verify that CsNAC17 specifically binds to the NACRS (CACG or CATGTG) elements of *CsRPM1* (Fig. 6e).

Since CsNAC17 and CsbHLH62 form heterodimers (Fig. 4), we suspected whether CsbHLH62 also regulates the expression of *CsRPM1* directly. Our Y1H test revealed that the bait strain, *ProCsRPM1*-pAbAi, grew normally on SD/Leu/AbA medium without pGADT7-CsbHLH62 plasmid transformation, but failed on SD/Leu/AbA^200^ medium (Fig. 6b). Additionally, dual-luciferase reporter assay also confirmed CsbHLH62’s inability to directly regulate *ProCsRPM1* (Fig. 6f). Interestingly, the regulation of *ProCsRPM1* by CsNAC17 was enhanced upon formation of heterodimers with CsbHLH62 (Fig. 6g and h).

### Reinforcement of *C. gloeosporioides* resistance in ‘LJ43’ leaves through transient overexpression of *CsNAC17* and *CsbHLH62*

**Figure 7:**
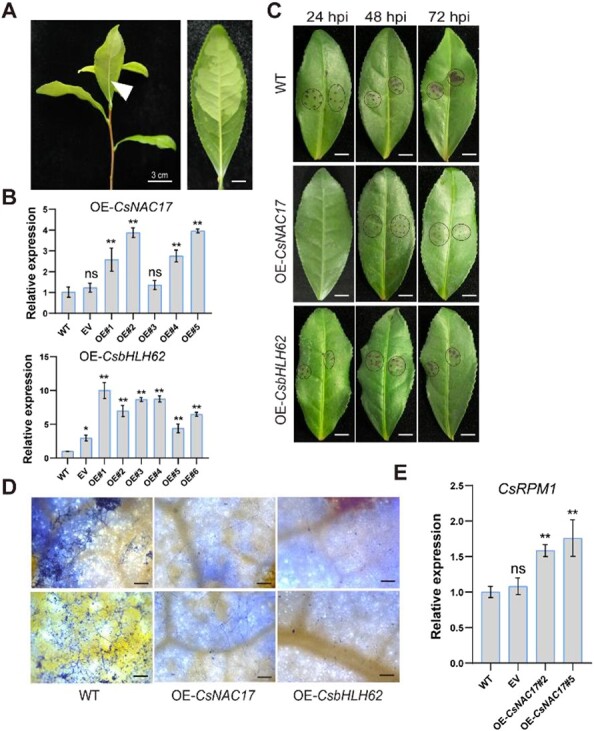
Transient overexpression of *CsNAC17* and *CsbHLH62* in tea plant leaves enhances resistance to anthracnose. (**A)** Petiole injection. Arrow for injection sites. (**B)** Relative expression of *CsNAC17* and *CsbHLH62* in leaves of ‘Longjing 43’ at 48 h after injection. RT-qPCR data with three independent replicates were introduced as mean values ± standard deviation (*, *P* <0.05; **, *P* <0.01). **(C)** Disease symptoms on ‘Longjing 43’ leaves after *C. gloeosporioides* inoculation at 24, 48, and 72 hpi. Black ellipse indicates the lesion. Scale bar = 2 cm. (**D)** Phenotypes of leaves of *CsNAC17* OE, *CsbHLH62* OE lines, and WT stained with trypan blue at 72 hpi. Scale bar = 200 μm. (**E)** Expression profiles of the *CsRPM1* in infiltrated leaves at 48 h after transient overexpression *CsNAC17*. OE#2, OE#5 were referred to the distinct CsNAC17 OE lines. RT-qPCR data with three independent replicates were introduced as mean values ± standard deviation (**, *P* <0.01).

Our objective was to explore the functional roles of *CsNAC17* and *CsbHLH62* in pathogen response by independently introducing these two genes into ‘LJ43’ leaves through transient transformation, followed by *C. gloeosporioides* inoculation (Fig. 7a). In comparison with untransformed leaves, *CsNAC17* and *CsbHLH62* transcripts increased more than 2-fold after infiltration. This observation confirms the successful overexpression of *CsNAC17* and *CsbHLH62* (Fig. 7b). *Colletotrichum gloeosporioides* caused significantly larger lesions on WT leaves than on CsNAC17 and CsbHLH62 OE plants, particularly at 72 hpi (Fig. 7c). At 72 hpi, WT leaves had a substantial number of *C. gloeosporioides* hyphae, while *CsNAC17* and *CsbHLH62* OE leaves had a few (Fig. 7d), respectively. In the OE-*CsNAC17* tea plants, *CsRPM1* transcripts were significantly higher at 48 h after injection compared to WT and empty vectors (EV) (Fig. 7e). The findings suggest that OE-*CsNAC17* and OE-*CsbHLH62* confer enhanced resistance against *C. gloeosporioides* in tea plants.

### Through transient silencing of *CsNAC17* and *CsbHLH62*, ‘ZC108’ leaves are less resistant to *C. gloeosporioides*

**Figure 8:**
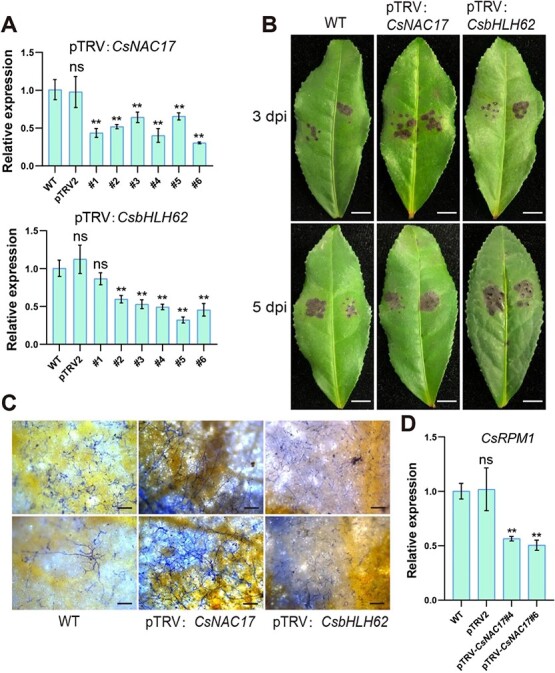
Transient silencing of *CsNAC17* and *CsbHLH62* in tea plant leaves reduces resistance to anthracnose. (**A)** Relative expression of *CsNAC17* and *CsbHLH62* in leaves of ‘Zhongcha 108’ at 48 h after injection. RT-qPCR data with three independent replicates were introduced as mean values ± standard deviation (**, *P* <0.01). (**B)** Disease symptoms on ‘Zhongcha 108’ leaves after *C. gloeosporioides* inoculation at 3 and 5 dpi. Scale bar = 2 cm. (**C)** Phenotypes of leaves of pTRV: *CsNAC1*, pTRV: *CsbHLH62* lines, and WT stained with trypan blue at 72 hpi. Scale bar = 200 μm. (**D)** Expression profiles of the *CsRPM1* at 48 h after transient silencing of *CsNAC17* in infiltrated leaves. #4, #6 were referred to the distinct CsNAC17 VIGS lines. RT-qPCR data with three independent replicates were introduced as mean values ± standard deviation (**, *P* <0.01).

Several species, including tea plants, have been broadly utilized the TRV-mediated VIGS system for researching gene function (Li et al., 2022; [32, 33]). VIGS approach achieved repression of *CsNAC17* and *CsbHLH62* transcripts in transiently transformed tea plant leaves in our study. The transcript levels analysis of *CsNAC17* and *CsbHLH62* at 48 h post-infiltration, revealed a significant reduction compared to the WT (Fig. 8a). After inoculation with *C. gloeosporioides*, the lesions on pTRV: *CsNAC17* and pTRV: *CsbHLH62* leaves exhibited significantly larger sizes contrasted with those on leaves of WT plants at 3 dpi (Fig. 8b). Notwithstanding, at 5 dpi, comparison of mutant and WT plants showed a decrease in the size of the lesions (Fig. 8b). Additionally, a higher abundance of hyphae was observed on pTRV: *CsNAC17* leaves compared to WT leaves at 3 dpi (Fig. 8c). Moreover, a significant lower transcript level of *CsRPM1* was found in pTRV: *CsNAC17* leaves than that in WT leaves (Fig. 8d).

Overall, the results supported the increased immunity of *CsNAC17* or *CsbHLH62* overexpressed leaves and the vulnerability of pTRV: *CsNAC17* or pTRV: *CsbHLH62* leaves to *C. gloeosporioides* infection, suggesting that *CsNAC17* and *CsbHLH62* played a crucial role in disease resistance.

### Reinforcement of *C. gloeosporioides* resistance in ‘LJ43’ leaves through transient overexpression of *CsRPM1*

**Figure 9:**
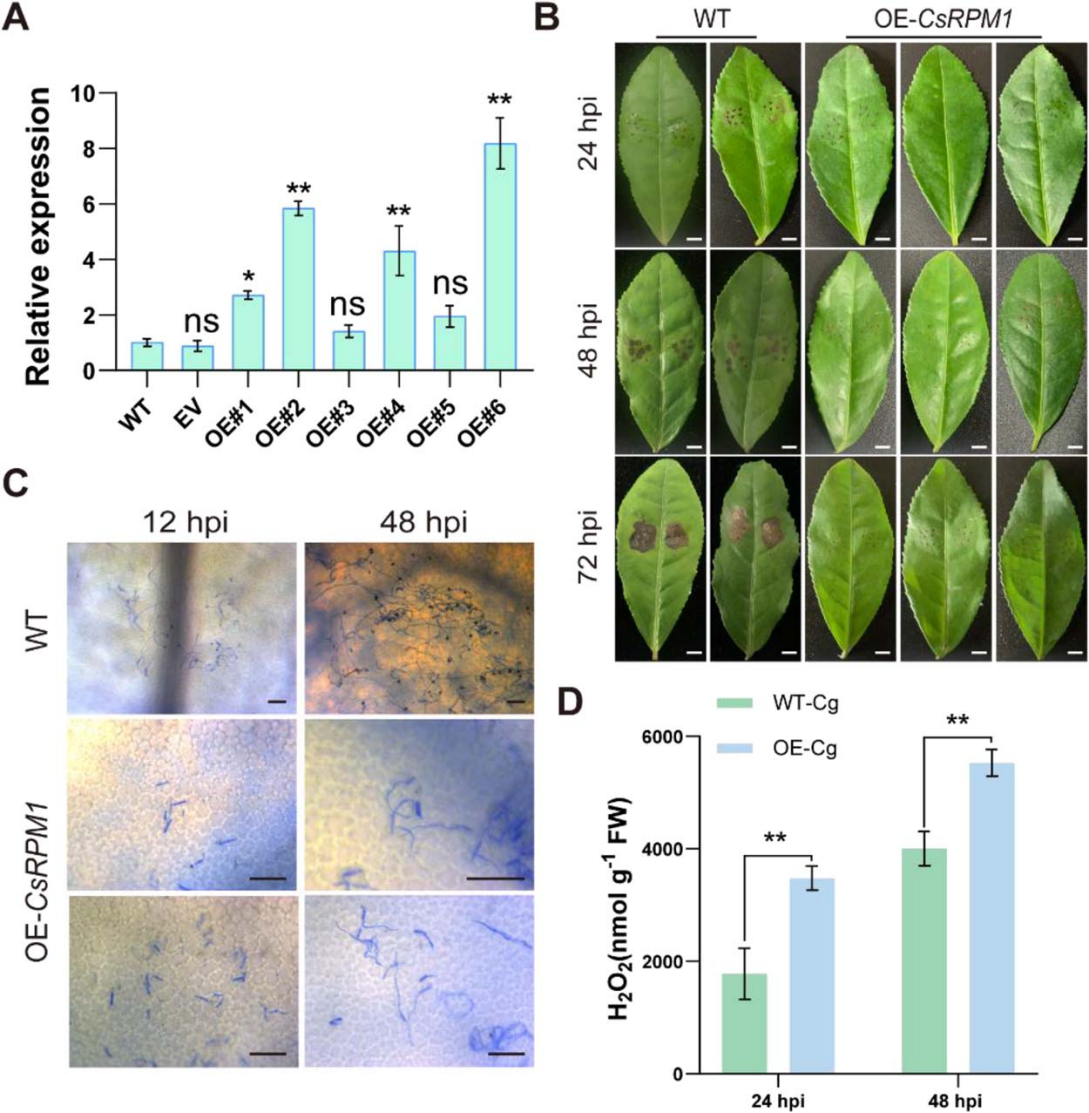
Transient overexpression of *CsRPM1* in tea plant leaves enhances resistance to anthracnose. (**A)** Relative expression of *CsRPM1* in leaves of ‘Longjing 43’ at 48 h after injection. RT-qPCR data with three independent replicates were introduced as mean values ± standard deviation (*, *P* <0.05; **, *P* <0.01). (**B)** Disease symptoms on ‘Longjing 43’ leaves after *C. gloeosporioides* inoculation 24, 48, and 72 hpi. Scale bar = 1 cm. (**C)** Phenotypes of leaves of *CsRPM1* OE lines and WT stained with trypan blue at 12 and 48 hpi. Scale bar = 50 μm. (**D)** Measurement of the H_2_O_2_ content in *CsRPM1* OE lines and WT leaves after infection with *C. gloeosporioides* at 24 and 48 hpi. Data were introduced as mean values ± standard deviation (**, *P* <0.01; *n* = 6).

We cloned the *CsRPM1* gene from ‘ZC108’ and constructed an overexpressing vector, which was transiently introduced into ‘LJ43’ leaves to investigate its role in anthracnose resistance. RT-qPCR data showed that *CsRPM1* was successfully expressed in tea plant leaves (Fig. 9a). Following *C. gloeosporioides* infection, the WT leaves began developing small lesions near the wound site within 24 hpi. As the infection progressed, the lesion gradually expanded to cover the entire wound area within 48–72 hpi. In contrast, the OE-*CsRPM1* leaves exhibit slightly visible, discernible lesions throughout the infection process (Fig. 9b). The trypan blue staining revealed hyphae formation on WT leaves by 12 hpi with *C. gloeosporioides*, whereas OE-*CsRPM1* leaves showed no spore germination. After 48 h of infection, WT exhibited substantial intertwined hyphae and appressoria, while OE-*CsRPM1* displayed only spore germination and shorter hyphae (Fig. 9c). Subsequently, H_2_O_2_ content analysis indicated that OE-*CsRPM1* leaves have a higher H_2_O_2_ accumulation than WT 24 hpi, with levels continuing to a continuous increase at 48 hpi (Fig. 9d). These findings suggest that OE-*CsRPM1* confers enhanced resistance against *C. gloeosporioides* in tea plants.

## Discussion

Anthracnose poses a significant threat to tea plants. To enhance anthracnose resistance, it is crucial to investigate key genes, unravel disease mechanisms, and employ molecular breeding for robust, disease-resistant *C. sinensis* varieties. Our study focused on the role of CsNAC17 and CsbHLH62 upon anthracnose infection. We demonstrated the interaction between CsNAC17 and CsbHLH62 by BiFC, Y2H, Co-immunoprecipitation (Co-IP), and Split-LUC assays. Overexpressing *CsNAC17* or *CsbHLH62* in tobacco and tea plant leaves both conferred increased resistance against anthracnose compared to WT (Fig. 3; Fig. 7c and d). Further, the transient suppression of *CsNAC17* or *CsbHLH62* in tea plant leaves compromised its resistance against anthracnose (Fig. 8b and c), indicating CsNAC17 and CsbHLH62 as positive regulators of immunity against *C. gloeosporioides*.

A critical aspect of PCD in plants involves HR induced by pathogen infection, representing a distinctive form of disease resistance. The oxidative burst intricately links to the allergic necrosis in host cells. Genetic resistance against wheat stripe rust is primarily controlled by the resistance genes, triggering ROS and HR to inhibit pathogens [34]. Upon wheat infection with stripe rust, the early accumulation of H_2_O_2_ was related with the hypersensitive cell death [35]. The novel protein promoter GP1 stimulates ROS and HR production, along with resistance-related genes and secondary metabolite accumulation, modulating plant defense responses [36]. HR and ROS were crucial in the resistance of tea plants to *Pestalotiopsis thea* in a concentrate on gray blight disease [37]. An accumulation of HR and H_2_O_2_ near the infection site was observed in the disease-resistant cultivar ‘ZC108’ against *C. fructicola* [38]. Notably, the H_2_O_2_ level in OE-*CsNAC17* transgenic tobacco exceeded that in WT, and trypan blue staining revealed significant HR accumulation around the mycelial infection site (Fig. 3d and e), suggesting HR and H_2_O_2_ as key factors contributing to anthracnose resistance.

The bHLH proteins play a crucial role in plant immunity through interactions with other proteins. Cotton GbbHLH177 interacts with a receptor-like kinase (RLK), GbSOBIR1, and can be phosphorylated by GbSOBIR1, contributing to resistance against *V. dahlia* [39]. MdbHLH093 activates salicylic acid signaling by interacting with MdMYB116 to enhance apple resistance against powdery mildew [19]. GmbHLH interacts with GmERF113, and transgenic plants overexpressing *GmERF113* exhibit enhanced resistance to soybean blight via positively regulating the expression of disease-related genes *PR1* and *PR10–1* [40]. The interaction between GhJAZ2 and GhbHLH171 leads to the inhibition of GhbHLH171’s transcriptional activity, thereby suppressing the JA-mediated defense response [41]. We discovered a novel interaction module between CsNAC17 and CsbHLH62 protein (Fig. 4). Additionally, the overexpression of *CsbHLH62* in tobacco and tea plant leaves enhanced resistance to *C. gloeosporioides* with increased H_2_O_2_ accumulation (Fig. 5, Fig. 7c and d). Conversely, transient suppression of *CsbHLH62* in tea plant leaves compromised the resistance of leaves against anthracnose (Fig. 8b and c).

Different transcription factors have been shown to activate R genes, thereby regulating plant resistance to pathogens. For example, *SlTGA9* modulates *Sl5R-1* expression in tomato plants, thereby affecting SA and JA signaling pathways and conferring resistance to tomato spotted wilt [42]. The regulation of the R gene by miRNA has also been demonstrated in several studies. Specifically, gma-miR1510 has been identified as a regulator that targets the GmTNL16, thereby enhancing resistance against *Phytophthora sojae* [43]. The NBS-LRR gene is targeted by miRNA482, prompting the cleavage of NBS-LRR mRNA and ensuing generation of secondary siRNAs. These secondary siRNAs can also co-regulate other disease resistance-related genes, collectively modulating plant disease resistance [44]. Our study showed that CsNAC17 can bind to the NACRS element of the *CsRPM1* promoter, with CsbHLH62 facilitating this binding (Fig. 6d–g). And it has been reported that G/E-box elements serve as the primary binding sites of bHLH TFs [45, 46], possibly explaining the lack of CsbHLH62 binding to the *CsRPM1* promoter. The R gene, being the pivotal gene family in plant disease resistance, exhibits a robust upregulation subsequent to pathogen infection, with its transcriptional level intricately linked to the plant’s protection reaction against pathogens [47, 48]. Previous reports indicate that transient silencing of the *CsRPM1* can negatively regulate tea plant resistance to *C. camelliae* [31]. In this study, transient overexpression of *CsRPM1* in ‘LJ43’ leaves enhanced resistance against *C. gloeosporioides* and elevated H_2_O_2_ levels (Fig. 9b–d). Our data revealed a physical association between CsNAC17 and CsbHLH62, synergistically enhancing *CsRPM1* expression and boosting resistance of tea plants against *C. gloeosporioides* (Fig. 6h).

*CsRPM1* and *CsNAC17* were significantly upregulated at 24–72 h after *C. gloeosporioides* infection, showing a similar expression pattern in both cultivars (Fig. 1e, Fig. 6a). Furthermore, *CsRPM1* expression was upregulated in *CsNAC17*-OE leaves (Fig. 7e), and downregulated in pTRV: *CsNAC17* leaves (Fig. 8d), suggesting that CsNAC17 directly targets the *CsRPM1* promoter, positively regulating its expression. We observed a significant upregulation in the transcription level of *CsbHLH62* at 72 h after *C. gloeosporioides* infection in ‘LJ43’ and ‘ZC108’ leaves, with minimal change at 24 and 48 hpi (Fig. 5b). This reinforces the finding that the CsbHLH62 alone maybe not bind to the *CsRPM1* promoter; rather, its interaction with CsNAC17 facilitates the expression of *CsRPM1*.

**Figure 10:**
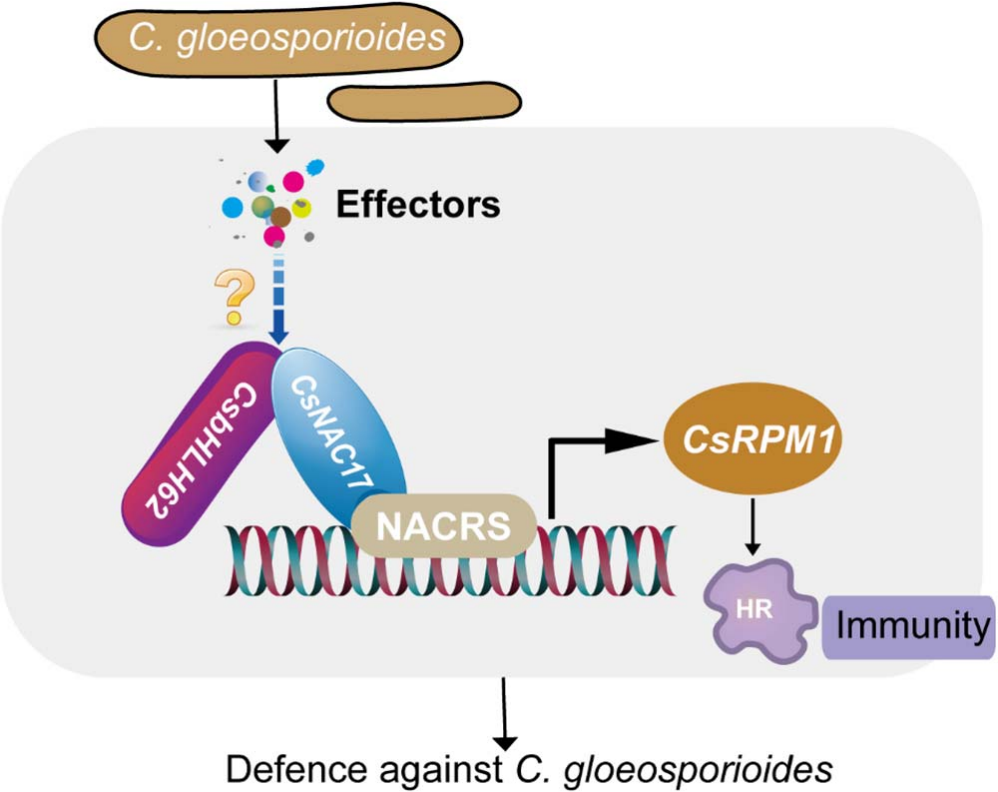
Schematic model for the role of CsNAC17 in resistance to anthracnose in tea plants. CsNAC17 binds to the NACRS elements in the *CsRPM1* promoter, thereby activating its transcription. CsbHLH62 enhances CsNAC17’s binding and activity on *CsRPM1*. *CsNAC17*–*CsbHLH62*–*CsRPM1* module induces a hypersensitivity reaction, thereby increasing the resistance to *C. gloeosporioides*.

In summary, we unraveled the role of *CsNAC17* in enhancing anthracnose resistance, as depicted in Fig. 10. The interaction between CsNAC17 and CsbHLH62 activates *CsRPM1*, triggering HR-associated cell death and H_2_O_2_ accumulation around the site of *C. gloeosporioides* infection, thereby enhancing the resistance of tea leaves to anthracnose. Consequently, *CsNAC17* and *CsbHLH62* serve as potential genetic resources for guiding germplasm selection and breeding. This study provides novel insights into how the bHLH family genes respond to pathogen invasion, and provides a basis for improving tea plant disease resistance through breeding or genome modification.

## Materials and Methods

### Plant materials

Two-year-old tea varieties ‘Zhongcha108 (ZC108)’ and ‘Longjing43 (LJ43)’ were cultivated under controlled conditions [29]. Both transgenic and non-transgenic plants were cultured in a plant incubator.

### Bioinformatic analyses

The physical and chemical properties were predicted by ProtParam (https://web.expasy.org/protparam/). Conserved domains were analyzed using the SMART website (http://smart.embl-heidelberg.de) and NCBI Conserved Domain Database. DNAMAN 7.0 software and MEGA5.0 were used to analyze sequence alignment of amino acids and perform phylogenetic analysis, respectively. The SWISS-MODEL website (https://swissmodel.expasy.org/interactive) predicted tertiary structure. ProtScale was used to assess hydrophilicity and hydrophobicity (https://web.expasy.org/protscale/).

### Subcellular localization

Using particular primers (Supplementary Table S1), the *CsNAC17* and *CsbHLH62* CDS (without stop codon) from the ‘ZC108’ leaf were intensified by PCR and subsequently introduced into the pCAMBIA2300 GFP vector. As previously described, *Agrobacterium tumefaciens* GV3101, which later converted these constructs into the tobacco leaf epidermis, was effectively injected with the expression vectors expressing CsNAC17-GFP, CsbHLH62-GFP, and the control empty vector [49]. After 48 h of incubation in dim light at 25°C, fluorescence was observed at 488 nm using a laser confocal microscope (ZEISS LSM 980 with Airyscan2, Germany). In order to locate the nucleus, mCherry red fluorescent protein is used.

### Generation of *CsNAC17*and *CsbHLH62* transgenic tobacco

For stable transformation of *N. benthamiana*, *A. tumefaciens* GV3101 carrying 35S-*CsNAC17*-*GFP* and 35S-*CsbHLH62*-*GFP* was utilized, following the previously described method [50]. A DNA extraction kit (Tiangen Biotech, China) was utilized to extract genomic DNA from tobacco leaves. The expression of *CsNAC17* and *CsbHLH62* was evaluated by PCR using primers (Supplementary Table S1) in transgenic tobacco lines. As a negative control, DNA from WT tobacco was used. RT-qPCR was utilized to detect expression of the *CsNAC17* and *CsbHLH62* genes.

### Transient expression in tea plant leaves and infection with *C. gloeosporioides*

In order to clone *CsNAC17*, *CsbHLH62*, and *CsRPM1* CDSs from ‘ZC108’ leaf, cDNA and gene-explicit primers were used (Supplementary Table S1). Amplicons were inserted into pCAMBIA2300GFP vector. After centrifugation, *A. tumefaciens* GV3101 cells containing recombinant plasmid were resuspended in an infiltration buffer as portrayed already [51]. The infiltration buffer was infused into the petioles of the second to third leaves in tea plant. As soon as the plants were infiltrated, they were grown at 24°C, 60%–70% relative humidity, and weak light for 24 h. Samples were then collected at 24, 48, and 72 h for subsequent experiments. WT was used as control experiments.

### Virus-induced gene silencing in *C. sinensis*

As previously described, VIGS was performed [52]. A 309 bp cDNA fragment of *CsNAC17* and a 351 bp cDNA fragment of *CsbHLH62* were inserted into the pTRV2 plasmid. *Agrobacterium tumefaciens* GV3101 carrying recombinant plasmid was cultured until OD_600_ = 1. Then, pTRV1 (OD_600_ = 1) was mixed with pTRV2-*CsCsNAC17* and pTRV2- *CsbHLH62* in a ratio of 1:1 (pTRV1+ pTRV2-*CsCsNAC17*; pTRV1+ pTRV2- *CsbHLH62*). The second to third leaves of the tea plant were injected by mixed infection solution through the vein. Samples were then collected at 24, 48, 72, and 120 h for subsequent experiments. WT was used as control experiments. In Supplementary Table S1, primers specific to these constructions are listed.

### Real-time quantitative polymerase chain reaction

As per the producer’s convention, the EASYspinPlus complex plant RNA kit (Aidlab Biotechnologies, China) was utilized to extract complete RNA from tea plant leaves under various treatments. HiScript® III RT SuperMix for qPCR with gDNA wiper (Vazyme, Nanjing, China) was used to synthesize the first-strand cDNA for RT-qPCR analysis. ChanQ®SYBR-qPCR Master Mix (Vazyme, Nanjing, China) was utilized for RT-qPCR analysis, with β-Actin serving as the reference gene [53]. All RT-qPCR assays were directed on the Bio-Rad CFX96 Touch real-time PCR system (Bio-Rad, USA). In Supplementary Table S2, primers are listed.

### Pathogen infection, histochemical staining

*Colletotrichum gloeosporioides* grew on PDA medium at 25°C in the dark for 10 days to enhance spore production. Subsequently, the spores were suspended in sterile water to assess the infection level, with a spore concentration of 2 × 10^5^ ml^−1^ for infection purposes. Plant materials used in the experiment were inoculated with *C. gloeosporioides*. The sterile water (CK) treatment was utilized as a control. The experimental designations are as follows: LJ43-Cg, LJ43-CK, ZC108-Cg, and ZC108-CK. The inoculation method is described as previously reported [29]. Trypan blue staining was conducted to observe plant cell death, fungal hyphae, and conidia, as mentioned earlier [54]. Aniline blue staining was conducted to observe the fungal development process [55]. DAB staining was used to evaluate the presence of H_2_O_2_. H_2_O_2_ was quantified as per the producer’s convention, and histochemical staining was observed under a stereo microscope and a light microscope (Olympus IX73, Japan).

### Yeast two-hybrid screening and assay

A protocol described in Coolaber’s Yeast Two-Hybrid Media Kit was utilized to investigate CsNAC17 transcriptional autoactivation. The positive control was co-transformed with pGBKT7–53 and pGADT7-T, and the negative control with pGBKT7-Lam and pGADT7-T.

The yeast library, constructed from the leaves and buds of ‘Zhongcha 108’, was used for the study. Employing the Yeast Two-Hybrid Media Kit screening system identifies the size of the insertion fragment within the yeast library, followed by sequencing to determine the genetic makeup of the insert. Eight positive clones were chosen for further analysis, with one candidate prey’s full-length CDS being cloned into ‘ZC108’ and embedded into the pGADT7 (AD) vector (Supplementary Table S1).

To study the interaction within yeast, the CsNAC17 and CsbHLH62 CDS was subcloned into the pGBKT7 and pGADT7 vector to express CsNAC17-BD and CsbHLH62-AD, respectively. Y2HGold cells were transfected with 150 ng ml^−1^ AbA and 50 g ml^−1^ X-α-Gal in SD/−Leu/−Trp/−Ade/-His medium, and blue coloration was used to screen for interactions.

### Bimolecular fluorescence complementation assay

The *CsNAC17* and *CsbHLH62* CDS (without stop codon) were cloned into pCV-nYFP and pCV-cYFP, respectively, to generate nYFP-CsNAC17 and cYFP-CsbHLH62. *Nicotiana benthamiana* leaves were transiently co-transfected with nYFP-CsNAC17 + cYFP, nYFP + cYFP-CsbHLH62, nYFP-CsNAC17 + cYFP-CsbHLH62, and mCherry as a nucleic marker [56]. Fluorescence was noticed utilizing a ZEISS LSM 800 confocal laser scanning microscope (Germany) at 48 h post-transfection. In Supplementary Table S1, primers specific to these constructions are listed.

### Split-luciferase assay

Tobacco leaves were coinjected with *Agrobacterium* GV3101 strains containing recombinant plasmids (nLUC-CsNAC17: cLUC-CsbHLH62 = 1:1, OD_600_ = 0.8) in the Split-LUC analysis. And containing recombinant plasmids nLUC-CsNAC17 and cLUC, nLUC and cLUC-CsbHLH62, nLUC and cLUC were mixed as a control. A CCD instrument (Princeton PIXIS 1024B, USA) was utilized to capture images of tobacco leaves 48 h post-application of luciferin (0.3 mg ml^-1^) [57]. In Supplementary Table S1, primers specific to these constructions are listed.

### Co-immunoprecipitation assay

To perform Co-IP, CsNAC17 and CsbHLH62 were inserted into pBWA(V)Hs-TMVΩ-4 × myc (CsNAC17-MYC) and pBWA(V)Hs-TMVΩ-3 × flag (CsbHLH62-FLAG), respectively. After transformation into *A. tumefaciens* GV3101 and infiltration into *N. benthamiana* leaves, protein extraction was performed as per the producer’s convention following a 48-h exposure to weak light (Thermo, USA). The Co-IP measure was directed by a formerly portrayed convention [58]. Target proteins were detected using Ig rabbit anti-MYC monoclonal antibody (Abclonal, AE070) and Ig mouse anti-FLAG monoclonal antibody (MBL, M185). IPKine horseradish peroxidase was used as the secondary antibody with goat anti-mouse IgG HCS. In Supplementary Table S1, primers specific to these constructions are listed.

### Yeast one-hybrid assay

The *CsRPM1* promoter sequence (1464 bp) was intensified and cloned into the pAbAi vector to generate a recombinant plasmid, pAbAi-*ProCsRPM1*. We prepared Y1HGold [*ProCsRPM1*-AbAi] competent cells using the Y1HGold-pAbAi Yeast One-Hybrid Library Screening kit from Coolaber (Beijing, China). Positive control was pGADT7-p53 transformed Y1HGold [p53-AbAi], while negative control was pGADT7 transformed Y1HGold [*ProCsRPM1*-AbAi].

CsNAC17-AD, as a prey strain, was cloned into pGADT7 and transformed into Y1HGold [*ProCsRPM1*-AbAi] bait strain. A solitary colony was chosen and cultured on SD/−Leu medium with 100 ng ml^−1^ Aureobasidin A to verify the positive interaction. The negative control used an unaltered pGADT7 vector transformed into the bait strains.

### Dual-luciferase assay

pGreenII 62-SK was utilized as an effector vector for CsNAC17 and CsbHLH62 (*Pro35S:CsNAC17*; *Pro35S*:*CsbHLH62*), while pGreenII0800-LUC was used as a reporter vector for CsRPM1 (*ProCsRPM1:Luc*) and three tandem copies of CATGTG (NACRS) (*3 × CATGTG:Luc*). In Supplementary Table S1, primers specific to these constructions are listed. All plasmids were independently changed into *Agrobacterium* GV3101. The *Agrobacterium* GV3101 carrying various reporter and effector plasmids, with combinations including *Pro35S:CsNAC17* + *ProCsRPM1:Luc*, *Pro35S:CsNAC17* + *Luc*, *Pro35S* + *ProCsRPM1:Luc*, *Pro35S* + *Luc*; *Pro35S:CsNAC17* + *3 × CATGTG:Luc*, *Pro35S:CsNAC17* + *Luc*, *Pro35S* + *3 × CATGTG:Luc*, *Pro35S* + *Luc*; *CsbHLH62 + ProCsRPM1:Luc*, *Pro35S:CsNAC17* + *ProCsRPM1:Luc*; *Pro35S:CsNAC17* + *Pro35S:CsbHLH62 + ProCsRPM1:Luc*, *Pro35S:CsNAC17 + ProCsRPM1:Luc* were co-penetrated into tobacco leaves. After 48 h, luciferin (0.3 mg ml^-1^) was uniformly applied to the tobacco leaves, which were then incubated in the dark for 5 min and imaged using a CCD instrument (Princeton PIXIS 1024B, USA). The Dual-Luciferase Reporter Assay System was utilized to quantify luciferase activity (Yeasen, Shanghai, China).

### EMSA

The CsNAC17 CDS was cloned into pGEX-6p-1-GST to form a recombinant plasmid CsNAC17-GST, which was transformed into *Escherichia coli* BL21 (DE3). After induction at 16°C, the protein was purified and recovered. The potential binding sites of CsNAC17 were extended by approximately 6 bp for synthesis of biotin-labeled hot probes, mutant probes with binding site mutations, and cold probes without biotin labeling. EMSA procedure was performed according to the LightShift Chemiluminescent EMSA Kit. The probe sequence is shown in Supplementary Table S1.

### Accession numbers

Sequence data in this article can be found in the Tea Plant Genome Annotation Project Database or GenBank data libraries under the following accession numbers: *CsNAC17*(GWHTACFB010158), *CsbHLH62*(GWHTACFB018089), *CsRPM1*(GWHTACFB018935), *CsActin*(CSA023174), *CsNAC100*-like(XP_028056118), *AcNAC*(PSR89671), *DlNAC100*(XP_052179391), *AdNAC*(QFG73549), *AdNAC6*(AZL19352), *MeNAC100*(XP_021614789), *TcNAC100*(XP_017972125), *VvNAC100*(XP_002284825).

## Supplementary Material

Web_Material_uhae295

## Data Availability

All relevant data are included in the article and its supporting materials.
